# Supplementary material for: Antibody persistence and neutralising activity in primary school students and staff: Prospective active surveillance, June to December 2020, England
Source: eClinicalMedicine. 2021 Sep 30;41:101150. doi: 10.1016/j.eclinm.2021.101150 (PMC8481203; doi:10.1016/j.eclinm.2021.101150)
Supplement: Supplementary file 1 [file mmc1.docx]

**Supplementary Material:** Antibody persistence and neutralising activity in primary school students and staff: prospective active surveillance, June to December 2020, England *Ireland et al.*

**Supplementary Figure 1: Correlation between live neutralising antibody titre and N-protein (a) and RBD (b) antibody quantitative results (COI) and live neutralising antibody titre in June (time 1) and July (time 2) (c) in students. Red lines denote the threshold values for reporting a positive result and brown line is where x=y.**

**Supplementary Figure 2: Violin Plot of live neutralising antibody titres by age (in years) on first N-protein positive sample (n=89). Red line denote the threshold values for reporting a positive result.**

**Supplementary Figure 1: Correlation between live neutralising antibody titre and N-protein (a) and RBD (b) antibody quantitative results (COI) and live neutralising antibody titre in June (time 1) and July (time 2) (c) in students. Red lines denote the threshold values for reporting a positive result and brown line is where x=y.**

1. *
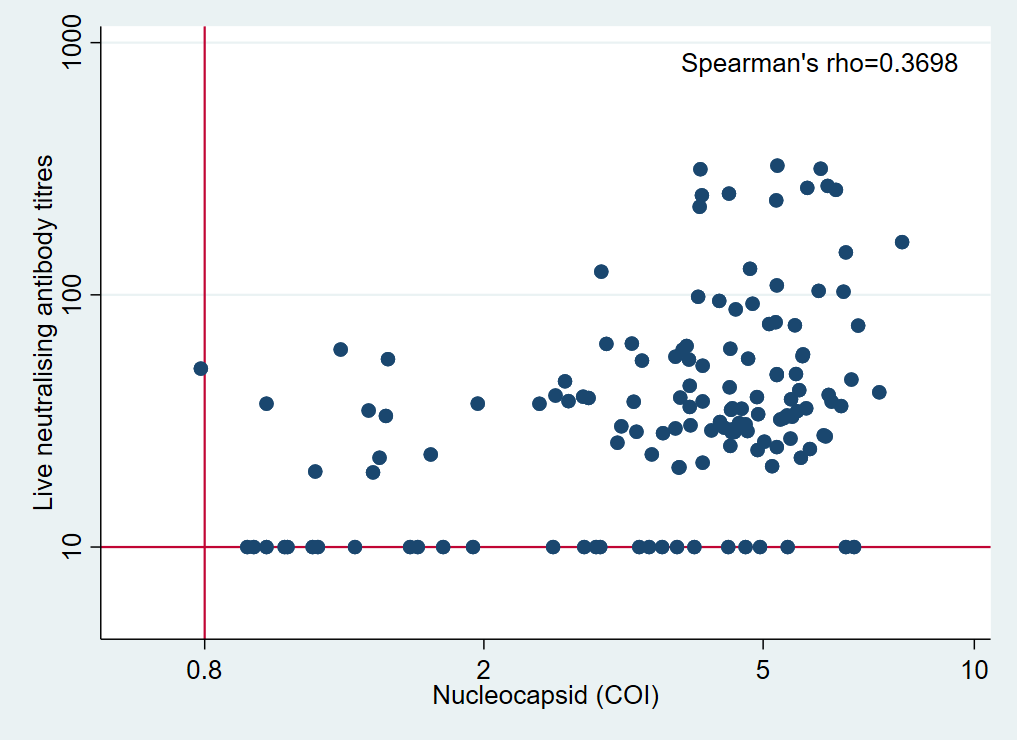
*
2. **
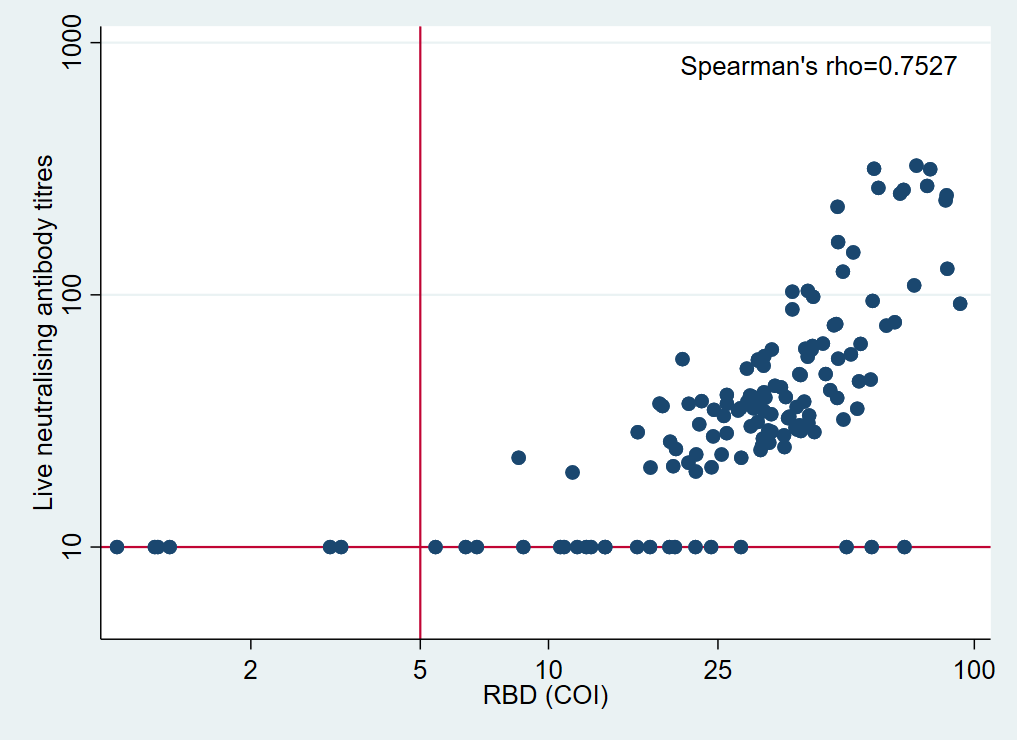
**
3.
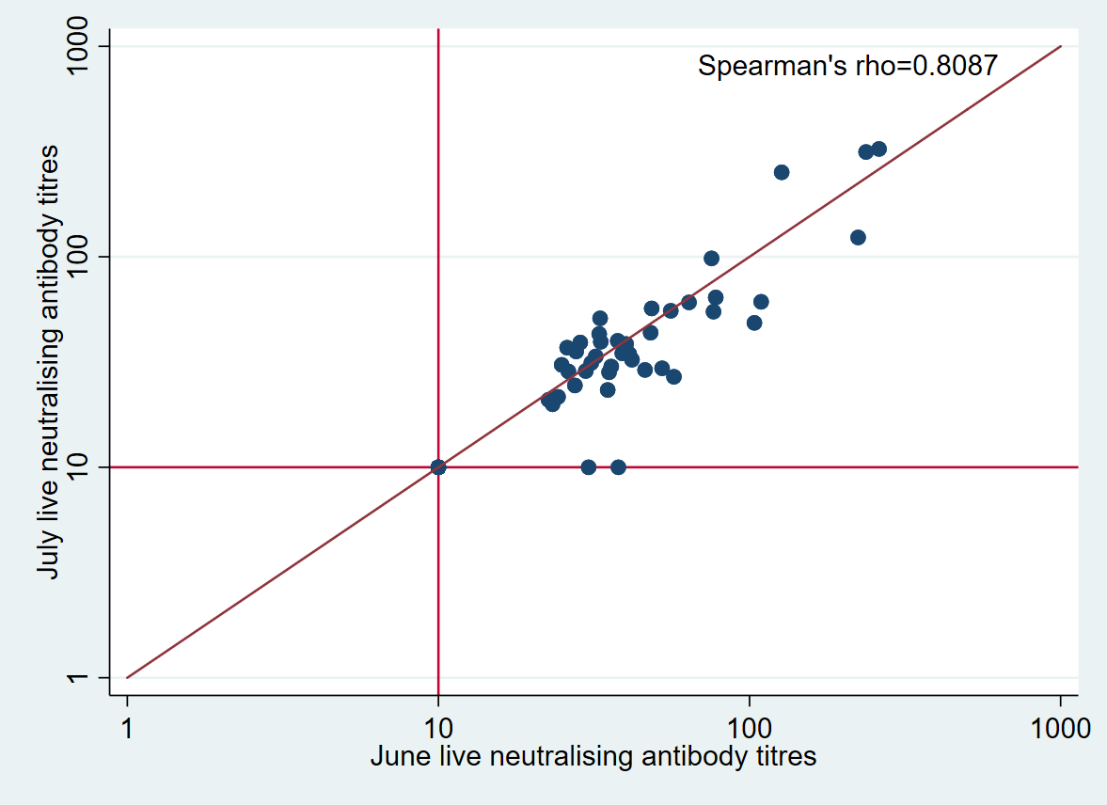


**Supplementary Figure 2: Violin Plot of live neutralising antibody titres by age (in years) on first N-protein positive sample (n=89). Red line denote the threshold values for reporting a positive result.**

***Within the violin plot: box = interquartile range (IQR), dot= median, vertical line= 1.5 IQR, curved line around boxplot= density plot***

**
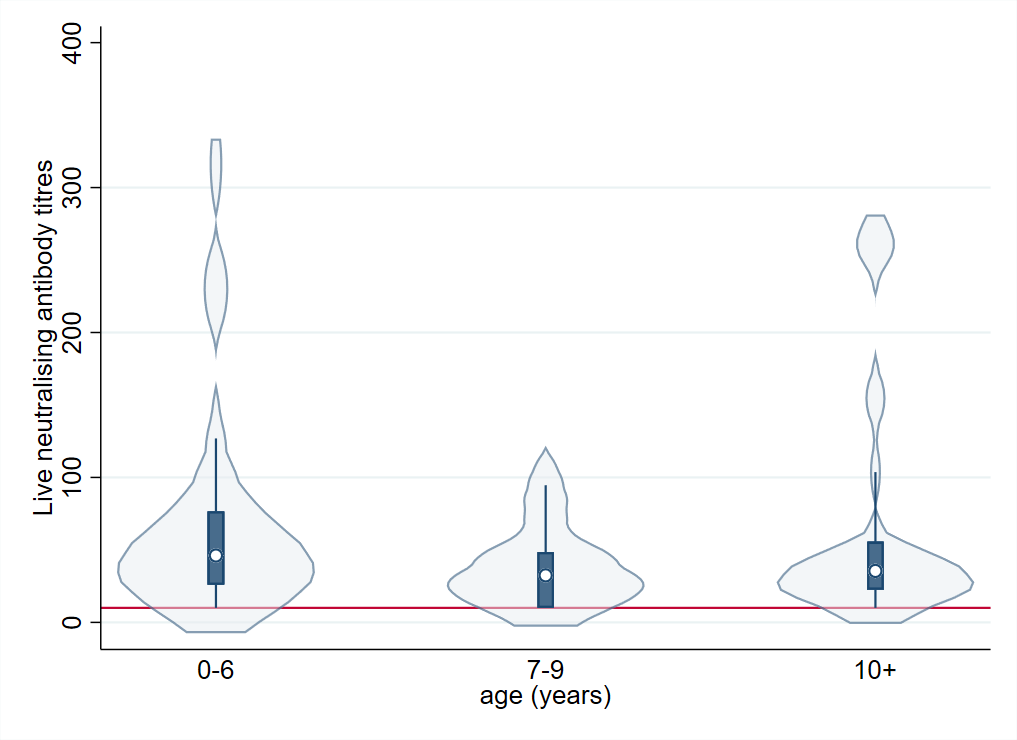
**
